# Probing Denaturation of Protein A via Surface-Enhanced Infrared Absorption Spectroscopy

**DOI:** 10.3390/bios12070530

**Published:** 2022-07-15

**Authors:** Valentina Di Meo, Massimo Moccia, Gennaro Sanità, Alessio Crescitelli, Annalisa Lamberti, Vincenzo Galdi, Ivo Rendina, Emanuela Esposito

**Affiliations:** 1 Institute of Applied Sciences and Intelligent Systems—Unit of Naples, National Research Council, 80131 Naples, Italy; valentina.dimeo@na.isasi.cnr.it (V.D.M.); gennaro.sanita@isasi.cnr.it (G.S.); alessio.crescitelli@cnr.it (A.C.); ivo.rendina@cnr.it (I.R.); 2 Fields & Waves Lab, Department of Engineering, University of Sannio, 82100 Benevento, Italy; massimo.moccia@unisannio.it (M.M.); vgaldi@unisannio.it (V.G.); 3 Department of Molecular Medicine and Medical Biotechnology, University of Naples Federico II, 80131 Naples, Italy; annalisa.lamberti@unina.it

**Keywords:** biosensors, metasurfaces, nanoplasmonics, surface-enhanced infrared absorption (SEIRA) spectroscopy, label-free detection

## Abstract

We apply surface-enhanced infrared absorption (SEIRA) spectroscopy to monitor the denaturation process of a surface-bound protein A monolayer. Our proposed platform relies on a plasmonic metasurface comprising different spatial subregions (“pixels”) that are engineered to exhibit different resonances covering the infrared region of the electromagnetic spectrum that is matched to the vibrational modes of the Amide groups. Specifically, we are able to determine changes in the Amide I and Amide II vibration coupled modes, by comparing the SEIRA reflectance spectra pertaining to the native state and a denatured state induced by a pH variation. In particular, we observe some evident red-shifts in the principal Amide I mode and the Amide II vibration coupled modes (attributable to the breaking of hydrogen bonds), which result in insurmountable barriers for refolding. Thanks to the strong field localization, and consequent enhancement of the light-matter interactions, our proposed sensing platform can operate with extremely small amounts of an analyte, with an estimated detection limit of about 3 femtomoles of molecules.

## 1. Introduction

Mid-infrared (Mid-IR) absorption spectroscopy is a powerful method for the structural determination of molecules in biological samples. With its spectral range of wavenumbers between 4000 and 400 cm^−1^ (i.e., wavelengths ranging from 2.5 to 25 μm), it enables the measurement of fundamental absorption bands deriving from vibrational states of most chemical bonds occurring in organic and biological molecules [1,2]. These molecular vibration frequencies essentially depend on the arrangement of the atoms, as well as the strength of the involved chemical bonds, which renders this technique a valuable tool for the investigation of proteins’ structure [3,4,5,6,7,8], molecular mechanisms underlying protein reactions [9,10], as well as protein folding, unfolding and misfolding [11,12]. Moreover, even for biological systems that are larger than proteins, Mid-IR absorption spectroscopy may still provide valuable information [13,14]. Further advantages include a broad application range (from small to large molecules), a high temporal resolution (down to 1 μs), a short measuring time, and a relatively low cost. In particular, Fourier-transform IR (FTIR) vibrational spectroscopy is a fast and routinely used chemical analytical technique suitable for the structural investigation of chemical compounds. It provides direct structural information on the composing functional groups of molecular species and the interactions they establish in all states of aggregation [15]. The absorption spectra also provide insightful information on the dynamics and environment in a univocal, non-destructive, and label-free way. For these reasons, mid-IR spectroscopy is used to identify substances in diverse applications spanning from pharmaceutical, safety, and food to forensic sciences. However, perhaps its stronger limitation is the very low molecular absorption cross-section of IR vibrations (σ_abs_ ≈ 10^−20^ cm^2^), which requires a large amount of material for accurate determinations. Accordingly, the spectroscopic characterization of minute amounts of analytes such as self-assembled monolayers (required for sensing applications) or biological membranes (naturally existing as single lipid bilayers) is not practical, in view of the extremely weak signals [15].

This fundamental limitation can be overcome in surface-enhanced IR absorption (SEIRA) spectroscopy [16,17,18], which relies on plasmonic metasurfaces (MSs). These structures, composed of two-dimensional (2-D) periodic arrays of sub-wavelength metallic elements (also called nanoantennas; NAs) laid on a dielectric substrate, can be suitably engineered to strongly localize electromagnetic (EM) waves (thereby enhancing their interaction with matter) [19], and achieve complete control of the wavefront properties [20,21,22,23], facilitating ultracompact device architectures for imaging [24,25] and biochemical sensing within spectral ranges from the ultraviolet (UV) to the mid-IR range [26,27,28]. The optical response of an MS can be tailored across the EM spectrum by varying the shape, size, and metal film thickness of each NA, as well as the array periodicity [29]. When MSs are resonantly coupled with the incoming EM radiation, the field can be strongly confined and enhanced around the NAs and into the gaps between them; this yields a several-order-of-magnitude amplification of the vibrational modes of nearby molecules, which in turn enables spectroscopic characterizations with unprecedented sensitivity [30,31]. In SEIRA spectroscopy experiments, to probe the presence of chemical and biological substances adsorbed, both the incident-radiation frequency and the molecular vibrational mode eigenfrequency must be matched with the MS resonance. As a consequence, the vibrational modes of the molecules are not directly observed as an absorption feature in the spectrum but appear instead as a modulation on top of the MS resonant line shape [16,17].

The key metric for SEIRA is the so-called “enhancement factor” (EF), which quantifies the amplification of the signal for a specific molecular vibrational mode. The enhancement mechanism stems from the interaction between the MS resonance and the molecular excitations, although the coupling mechanisms are not fully understood. However, it is well-established that the enhanced vibrational signal scales with the field intensity [17]. From the EM design viewpoint, the challenge is to judiciously exploit the geometrical and constitutive degrees of freedom available (NA shape and size, array period, substrate refractive index) in order to attain a desired resonance wavelength [32,33,34,35]. Moreover, especially for low concentrations of the analyte, it is crucial that the field hotspots occur consistently and homogeneously across the MS, so that the probability of enhanced light-matter interaction is maximized; this requires reliable and reproducible nanofabrication approaches. For a recent comprehensive review, the reader is referred to [36] and references therein.

In a series of ongoing studies [37,38,39], we have focused on the optimized design and fabrication of plasmonic MSs for SEIRA spectroscopy. For the design, we have developed some accurate and effective forward- and inverse-modeling strategies for selecting the NA shape and spatial arrangement, aimed at maximizing the detection sensitivity even for extremely small molecules. For the fabrication, we have developed a manufacturing process that is reproducible, highly homogeneous (<10% variability), versatile, fast, and provides reusable devices [37,38,39]. Specifically, our fabricated sensors were able to determine amounts of immobilized small molecules (molecular weight (MW) < 300 g/mol) equal to 0.7 fmoles, with a SEIRA EF of [37]. We also succeeded in detecting 25-hydroxyvitamin D (25(OH)D3—calcifediol) via FTIR spectroscopy, at a concentration as low as 86 pmol/L, and an amount of immobilized small molecules of 25(OH)D3 monohydrate (MW: 418.65 g/mol) as low as 4.31 amoles over an area of 100 × 100 μm^2^ [38]. In this case, we adopted an innovative design based on a multiresonant MS made of different pixels (covering an area of 500 × 500 μm^2^) each one designed to match a specific vibrational band, so that the whole MS could recognize different vibrational bands occurring within different parts of the MIR spectrum, from the region of functional groups to that of the fingerprint. By exploiting a similar approach, together with a specific bio-functionalization, we were also able to attain univocal and label-free recognition of complementary deoxyribonucleic acid (DNA) fragments in concentrations as low as 50 fM, i.e., well below the value attained by standard methods, with additional advantages in terms of processing time [39].

In this work, we take a further step and apply SEIRA spectroscopy to probe the denaturation of a protein monolayer. The native state of proteins is characterized by a highly ordered 3-D conformation. In this state, proteins exhibit a closely compact core and a highly folded structure and are biologically active [40,41]. However, this structure is relatively stable, and hence even small changes in the chemical–physical environment can induce unfolding and subsequently a denatured state. Proteins repeatedly unfold and refold during their lifetime, and the folding/unfolding process is crucial since biological health depends on its correct functioning. Often, perturbation processes, such as temperature and pH variations, are induced on protein samples to study their functional, aggregation, and folding/unfolding mechanisms [42,43]. Typical experimental methods for monitoring the protein unfolding [44], such as nuclear magnetic resonance (NMR) spectroscopy [45], are expensive and complicated to use. For example, the Amide backbone hydrogen/deuterium exchange (HDX) can be probed via NMR or mass spectrometry [46], or circular dichroism spectroscopy [47]. In addition, conformational changes of proteins containing chromophoric groups can be studied by UV-visible absorption spectroscopy [47]. Against this background, SEIRA spectroscopy can provide real-time, high-sensitivity, label-free monitoring in a simpler and cheaper fashion. The advantage of using SEIRA is that monolayers and/or extremely small quantities of molecules can be studied at very low concentrations, thanks to the large surface EF, and it is possible to detect the vibrational bands of few molecules selectively bound to the NA surface, thanks to precise methods of surface chemistry.

In what follows, we demonstrate the monitoring of a denaturation biological process obtained with a chemical denaturant that induces a pH variation, over a dehydrated monolayer of protein A (PA) as a sample. PA is a 49 kDa surface protein produced by staphylococcus aureus and encoded by the spa gene. PA binds with high affinity to the constant fragment of human immunoglobulins, and to Immunoglobulin G (IgG) from various species [48], owing to the presence of five IgG-binding domains folded into a three-helix bundle [49]. In this study, PA was chosen as a “model protein” to demonstrate the ability of our proposed system to detect protein denaturation without a complex analysis and in a very short time.

Denaturants can affect the chemistry of the amino acids and induce the unfolding of the polypeptide chain. We can determine changes in the Amide I and Amide II vibration coupled modes by comparing the SEIRA reflectance spectra pertaining to the native and denatured states. We estimate a wavenumber red-shift of 9 cm^−1^ for the principal Amide I mode, and of 10 cm^−1^ for the Amide II vibration coupled modes. The EF of our proposed MSs, within the spectral range of Amide I and II, varies between 3 × 10^4^ and 7 × 10^4^, which pushes the sensitivity to a limit of detection of 3.33 femtomoles of molecules.

## 2. Materials and Methods

The development of our proposed sensing platform revolves around four main lines: EM modeling and design, fabrication, biofunctionalization, and IR characterization.

### 2.1. Modeling and Design

**Figure 1:**
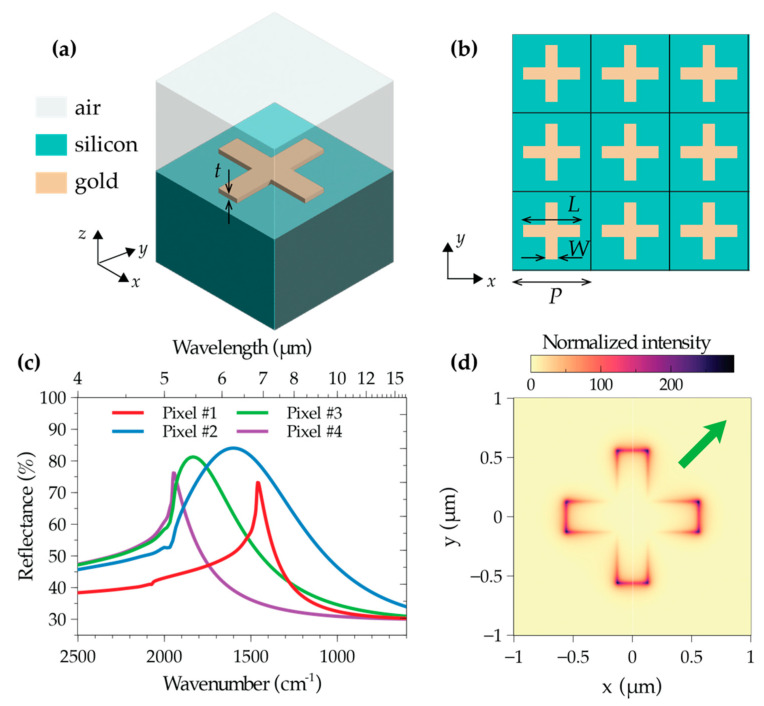
MS modeling and design. (**a**,**b**) Schematic of unit-cell and periodic array (top view), respectively. (**c**) Numerically computed reflectance responses, for plane-wave illumination with normal incidence and *x*-polarized electric field, and four representative parameter configurations (pixel #1: *p* = 2.0 µm, *L* = 1.117 µm, *W* = 0.255 µm; pixel #2: *p* = 1.5 µm, *L* = 1.247 µm, *W* = 0.267 µm; pixel #3: *p* = 1.5 µm, *L* = 1.027 µm, *W* = 0.270 µm; pixel #4: *p* = 1.5 µm, *L* = 0.843 µm, *W* = 0.250 µm). (**d**) Intensity distribution (electric field, normalized with respect to the impinging one) within a unit-cell, for pixel #1, computed 10 nm away from the nanoantenna at the resonant wavenumber 1457 cm^−1^. The thick green arrow indicates the polarization of the impinging electric field (not aligned to the cross arms, for better visualization).

For the EM modeling and design of the multiresonant plasmonic MSs, we utilize the finite-element-based commercial software package COMSOL Multiphysics v. 5.1 [50]. The basic geometry is schematized in Figure 1a,b: we consider cross-shaped gold NAs (of thickness *t* = 50 nm, arm length *L*, and arm width *W*) arranged on a 2-D periodic square grid (of period *P*), laid on a silicon substrate, embedded in air. Such geometry has already been successfully utilized in previous studies [37,38,39], demonstrating very good performance in terms of field enhancement and robustness with respect to polarization. Assuming, for simplicity, infinite periodicity in the transverse plane and normally incident plane-wave illumination from the air region, we can reduce our study to a single 3-D unit-cell (shown in Figure 1a), with the lateral walls terminated with periodic boundary conditions. Moreover, for the air and substrate regions, we assume a finite thickness (10 µm and 5 µm, respectively), and apply a port-type and a perfectly matched layer, respectively, for terminations. For the constitutive parameters, we assume an electrical conductivity σ = 15 × 10^6^ S/m for gold [51], and the refractive index in [52] for silicon. Finally, we discretize the resulting computational domain by relying on standard meshing (which yields ~3 million degrees of freedom) and apply the standard MUMPS solver with default parameters [50].

Our design procedure relies on a preliminary parametric study, in which we extensively explore the geometrical parameter space and identify possible resonances of interest. As an example, Figure 1c shows some representative numerical simulations of reflectance responses covering the spectral range of interest. We highlight that the abrupt changes of slopes that can be observed at specific wavenumbers are attributable to the well-known Rayleigh–Wood anomalies for the transmitted field [53]. As a general observation, we note that a particularly favorable parameter regime is *P/λ_res_* ~0.5 (with *λ_res_* denoting the resonant wavelength), where collective effects tend to favor sharper resonances [54]. Figure 1d shows a representative resonant-field distribution, from which we observe a strong field enhancement (up to a factor ~300) with hotspots at the arm tips, as typical for plasmonic NAs.

For the inverse design, i.e., the synthesis of a resonance with desired position and linewidth, we start from a coarse initial guess from the computed codebooks, and progressively fine-tune the parameters.

### 2.2. Fabrication

**Figure 2:**
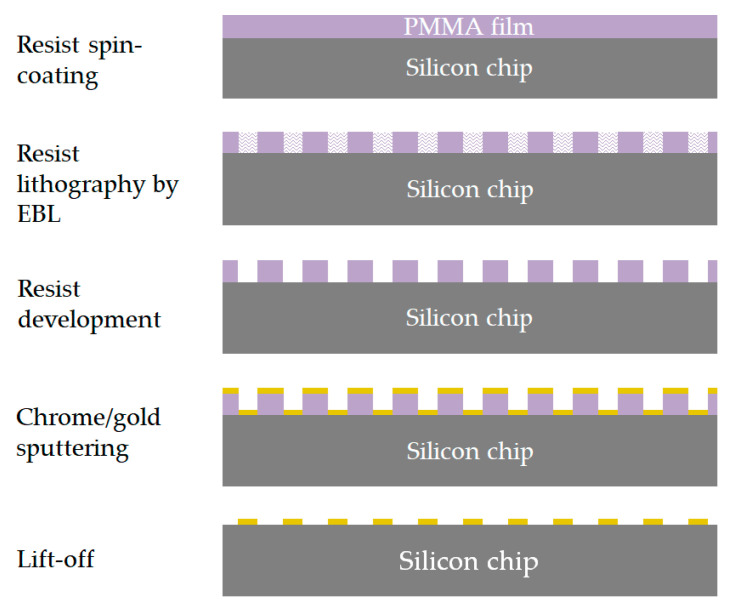
Schematic illustration of the fabrication process.

**Figure 3:**
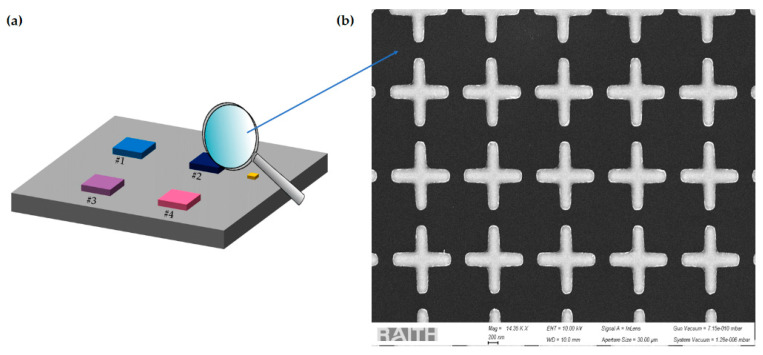
(**a**) Schematic of the proposed MS. Pixels are identified by a unique number; (**b**) SEM image of pixel #2.

The fabrication of the designed multiresonant MSs requires precise control of the NA shape, size, and spacing in order to attain a reliable and reproducible tuning of the plasmonic resonances. Among the possible techniques able to realize ordered arrays of metallic nanostructures, such as direct laser writing [55], nanoimprint lithography [56], laser interference lithography [57], and electron beam lithography (EBL), the latter is one of the most employed due to its high reproducibility and flexibility of use [37]. For these reasons, the proposed multiresonant MS is fabricated on a 10 × 10 mm^2^ float-zone silicon chip by means of EBL and lift-off processes, as schematically illustrated in Figure 2. The MS consists of four separate pixels, each one representing a 2-D array of cross-shaped NAs; such symmetrical shape renders the optical response robust with respect to the polarization of the impinging light and to the random orientation of the target molecules. The pixels (each covering an area of 500 × 500 µm^2^) are characterized by different designs (summarized in Table 1), in order to exhibit resonances covering the wavenumber range 1500–2100 cm^−1^ (i.e., 4.8–6.7 µm in wavelength). Close to the pixels, an unpatterned gold mirror is deposited, which provides a reference reflectance response used for normalization. In detail, a positive tone electron beam resist is spin-coated onto the silicon die, and then the desired pattern is transferred into the resist layer by means of EBL (with an acceleration voltage of 10 kV, a numerical aperture of 10 µm, and an energy dose of 100 µC/cm^2^) and the subsequent resist development. Finally, a 5 nm thick chromium film (acting as a buffer layer) and a 50 nm thick gold layer are sputtered onto the patterned resist; after the lift-off process, the desired MS is obtained. Figure 3a shows a schematic of the proposed MS, while Figure 3b shows the scanning electron microscope (SEM) images of a representative pixel (#2).

### 2.3. Biological Assay Preparation

#### 2.3.1. Bioconjugation of MS Pixels with PA

**Figure 4:**
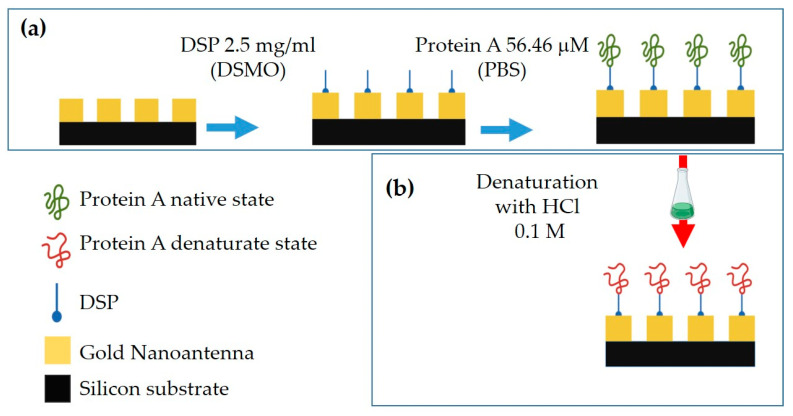
Schematic of PA binding on gold Nas and of denaturation process: (**a**) Binding of PA on gold surface by using DSP molecule; (**b**) PA denaturation process by incubation in HCl 0.1 M.

All pixels are biofunctionalized with PA by using dithiobis(succinimidyl propionate) (DSP), Lomant’s Reagent. Specifically, the pixels are washed with dimethyl sulfoxide (DMSO), isopropyl alcohol, and bi-distilled water for 5′ under stirring. After drying by N_2_ fluxed, the gold surface is incubated with DSP 2 mg/mL in DMSO for 60′ at RT. After extensive washing with DMSO, bi-distilled water, and phosphate-buffered saline (PBS) 1×, the surface is incubated with PA 56.46 µM in PBS overnight at 4 °C. Next, after extensive washing with PBS 1× and bi-distilled water, the pixels are dried by N_2_ fluxed. Figure 4a shows a schematic of this process.

#### 2.3.2. PA Denaturation Protocol

To denature the PA bonded to the MS, all pixels are incubated with HCl solution (0.1 M) for 60′ at RT. After extensive washing with PBS 1× and bi-distilled water, the surface is dried by N_2_ fluxed. Figure 4b shows a schematic of this process.

### 2.4. IR Characterization

The MIR spectroscopy of our plasmonic pixeled MSs is carried out by using a Thermo-Nicholet NEXUS Continuum XL (Thermo Scientific, Waltham, MA, USA) equipped with a mercury cadmium telluride nitrogen cooled detector and a microscope system with a 10× optical or 15× IR magnification. The measurement area is set to 100 × 100 µm^2^ thanks to knife-edge apertures. All acquired reflectance spectra are automatically normalized to the reference spectrum of the gold mirror deposited on the same substrate (see Figure 3a). The measurements are performed with a spectral resolution of 4 cm^−1^ by collecting, at room temperature, the reflected signal within the spectral region 4000–400 cm^−1^, by using 128 scans with 5 s acquisition time for each spectrum.

## 3. Results and Discussion

**Figure 5:**
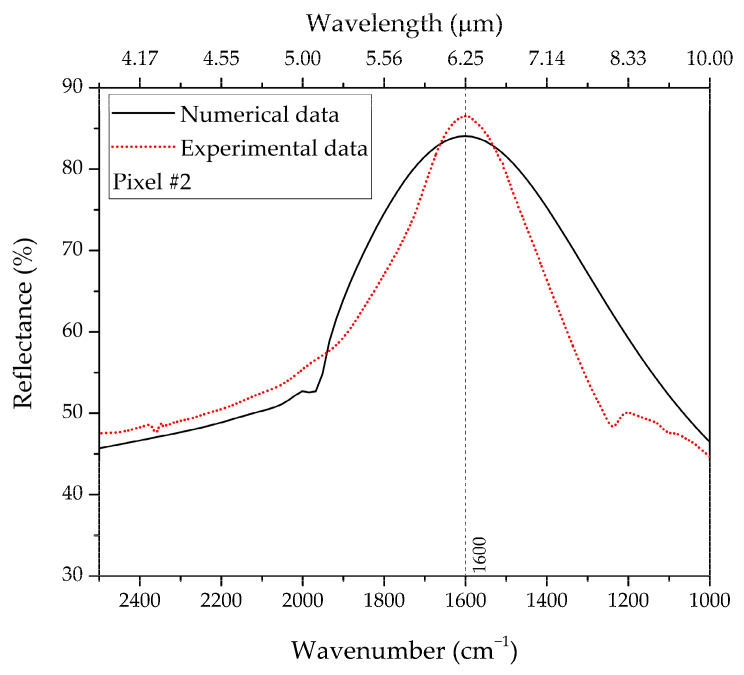
Comparison between numerical (black-solid curve) and experimental (red-dotted) reflectance responses pertaining to pixel #2 for naked NAs (no molecules adsorbed).

As a preliminary validation step, we compare the pixels’ experimental resonances with the simulated ones. Figure 5 shows, as an example, the comparison between the simulated and experimental reflectance resonant spectra pertaining to pixel #2.

**Figure 6:**
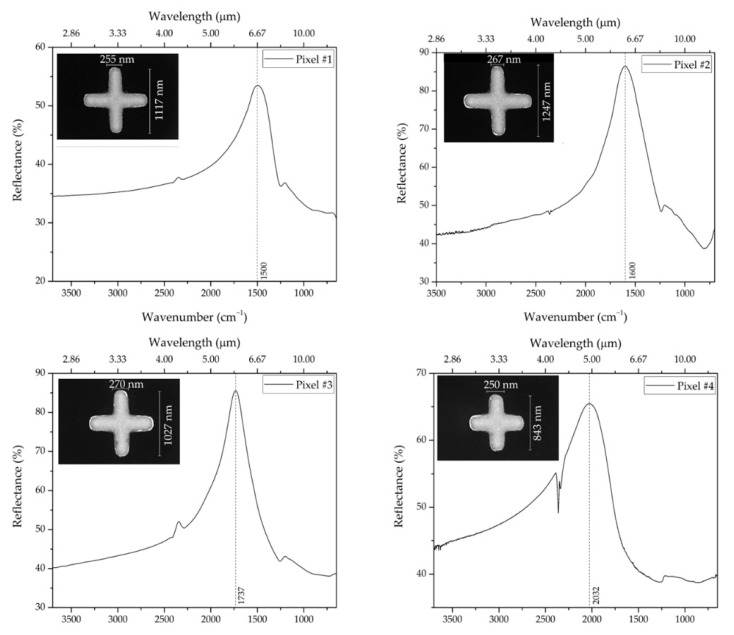
Experimental reflectance responses pertaining to all pixels for naked NAs (no molecules adsorbed); each inset shows the corresponding NA SEM image and dimensions.

From the experimental data, we observe a plasmonic resonance peak (~85% reflectance) at a wavenumber of 1600 cm^−1^, in good agreement with the numerical one obtained for the geometrical parameters reported in Table 1. This validates our design procedure of the MS pixels. The pronounced linewidth of the resonance is not a disadvantage, since it enables to match the entire vibrational band expected in that specific IR spectral range (Amide spectral region, in the example). Figure 6 shows the resonant responses of the four pixels, with the corresponding NA SEM images and dimensions shown in the insets. As it can be observed, each pixel is able to detect different vibrational bands covering a wide area from ~1200 to 2200 cm^−1^. From the comparison between the experimental and numerically computed resonant wavenumbers for the four pixels (Table 1), we observe a generally good agreement, with discrepancies up to ~6%, which can be attributed to fabrication tolerances and model uncertainties in the constitutive parameters.

**Figure 7:**
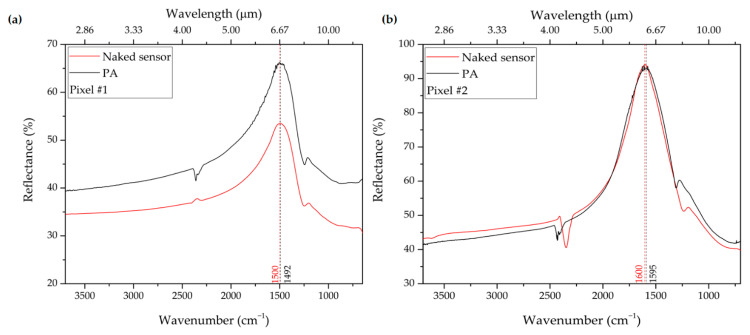
Red-shift of the resonance after the functionalization of (**a**) pixel #1 and (**b**) pixel #2.

Once the spectral regions of specific biological interest are identified, the entire MS is functionalized. To verify the binding of the coupling molecules to the NA surface, we monitor the red-shift of the resonance for each pixel. As a representative example, the response pertaining to pixel #1 is shown in Figure 7.

In this case, due to the PA monolayer linked to the NA surface, we observe a red-shift of 8 cm^−1^ for pixel #1 and of 5 cm^−1^ for pixel #2. During the functionalization, a molecule of DSP binds to each gold atom exposed on the surface; subsequently, a molecule of PA binds to each molecule of DSP. The SEIRA spectra are acquired on dehydrated protein monolayers.

In proteins, the most present functional groups are Amide groups; the two more relevant Amide vibrations are: Amide I and Amide II. The Amide I vibration consists primarily of the C=O stretching vibration of the Amide group, with weaker contributions from the Amide C−N stretching and the N−H bending. Amide I vibrations of backbone Amide groups are not independent but mix into coupled modes [58,59,60]. In polypeptides and proteins dominated by helical structures, the Amide I vibration is centered around 1665–1635 cm^−1^ and, when the β-sheet content is dominant, at 1640–1615 cm^−1^. Fitting methods place the Amide I bands within the spectral range 1700–1620 cm^−1^ [46].

The Amide II vibration is mainly due to the out-of-phase combination of the N−H in-plane bend and the C−N stretching vibration. The resulting coupled Amide II modes bands cover the spectral range 1560–1500 cm^−1^. Helical structures are grouped around 1555–1545 cm^−1^, and β-sheets around 1535–1525 cm^−1^ [61,62,63].

Proteins in their native and biologically active state exhibit a well-ordered and usually folded structure. In the absence of denaturing circumstances, the 3-D structure of many proteins is compact and ordered, with a tightly packed core. The agents that can denature a protein are either chemical or physical, or even an altered thermodynamic state. They produce an unfolding of the polypeptide chain with the disruption of noncovalent bonds between amino acids, while the covalent structure remains intact. Furthermore, the folded protein structure is very susceptible to chemical and/or physical variations, so even small alterations of their solvents can cause unwanted unfolding and hence denaturation. Therefore, the availability of a real-time and unlabeled verification method is crucial, and SEIRA spectroscopy can be a valid tool in this respect.

We monitor the state of the surface-bound protein monolayer through the comparison of the reflectance absorption SEIRA spectra between the native denatured states for each pixel. In particular, pixel #1 and pixel #2 are designed to match the Amide group of the polypeptide backbone vibrations.

**Figure 8:**
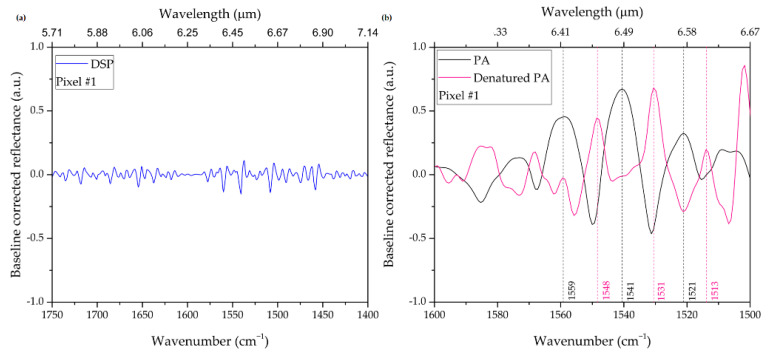
Baseline-corrected reflectance SEIRA spectrum pertaining to Pixel #1. (**a**) NAs functionalized with DSP molecules. (**b**) NAs with dehydrated monolayer of PA in the native (black curve) and denatured (purple curve) states.

**Figure 9:**
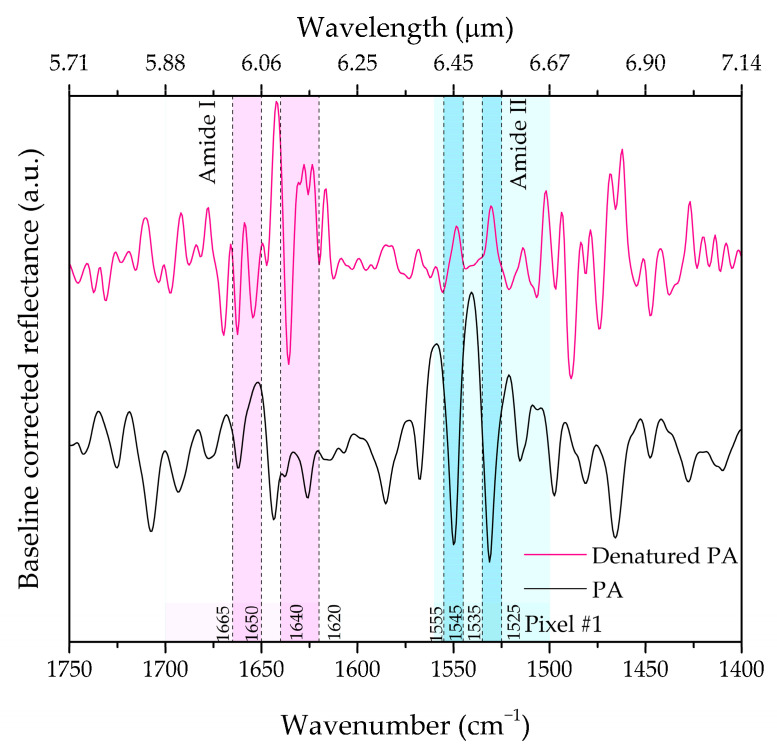
Pixel #1 baseline-corrected reflectance SEIRA spectrum of a dehydrated monolayer of PA (black curve) and of the denatured state (purple curve) after the immersion in HCl at 0.1 M with a PH = 1. Note the shift of the bands pertaining to Amide I (pink area) and Amide II (green area). The spectral region around 1665–1650 cm^−1^ pertains to the helical structure Amide I vibration, while the one around 1640–1620 cm^−1^ pertains to σ-sheet. The spectral region around 1555–1545 cm^−1^ pertains to the helical structure Amide II vibration, while the one around 1535–1525 cm^−1^ pertains to β-sheets.

With reference to pixel #1, Figure 8a shows the SEIRA spectrum pertaining to the NAs with the sole functionalization of the DSP, whereas Figure 8b shows the spectra in the presence of protein A in the native state (black curve) and in the denatured state (red curve). Specifically, the baseline-corrected reflectance SEIRA spectra are shown as a function of the wavelength (top *x*-axis) and wavenumber (bottom *x*-axis), with an indication of the relevant peaks and bands. In our experiment, we denature the PA monolayer by immersing the substrate in a solution with pH = 1, following the protocol described in Section 2.3.2. We subsequently re-acquire the IR spectrum under the same conditions as before denaturation. For a clearer interpretation, the SEIRA spectra of the native PA and its denatured state are also shown in Figure 9, overlayed with the Amide I and II regions (shaded areas). We observe that the peaks appearing in the Amide I and II regions do not show up in the same environmental conditions in the absence of protein A (i.e., with the sole functionalization of the DSP, cf. Figure 8b). Hence, we are able to observe some spectral features that are clearly attributable to protein A, although there is significant room for optimization of the signal-to-noise ratio in our spectra. We are currently working on improving this aspect, which will be the subject of a forthcoming study.

In particular, as shown in Figure 9, we can determine changes in the Amide I and Amide II vibration coupled modes. In the region 1665–1650 cm^−1^, we estimate a red-shift of 9 cm^−1^ for the Amide I band, which is frequently interpreted in terms of structural changes of α-helical structures. Moreover, we estimate a red-shift of 10 cm^−1^ for the Amide II vibration coupled modes [44]. These red-shifts are mainly due to the breaking of hydrogen bonds, which can result in insurmountable barriers to refolding [44].

The EF provided by our multiresonant MS can be estimated by relying on a well-established model [37]. With reference to the Amide modes, for our designed pixels, we obtain values ranging from 3 × 10^4^ to 7 × 10^4^, which allow revealing the vibrations of extremely small quantities of molecules. Since the field of view of the IR microscope is limited to an area of 100 × 100 µm^2^, we can evaluate the number of molecules excited by the IR radiation from which we detect the vibrational modes; this turns out to be 3.33 fmol. In fact, this number is in excess, since there are surface selection rules for which not all theoretically predicted vibrational modes are observed in the experimental spectra, but only those chemical bonds whose orientation is perpendicular to the gold NA surface [64].

## 4. Conclusions

In this study, we have reported the monitoring of a denaturation process occurring to a PA monolayer, by utilizing a plasmonic MS as the substrate in SEIRA spectroscopy. Our proposed platform allows the monitoring of the whole Amide spectral region 1500–1700 cm^−1^, thanks to a multiresonant pixeled design. Specifically, each pixel is able to detect different vibrational bands, with the entire MS covering a broad spectral range from ~1200 to 2200 cm^−1^. We have demonstrated real-time and label-free monitoring of the surface-bound monoloayer of PA via FTIR spectrometry, by comparing the SEIRA spectra of the native and denatured states. We can determine changes in the Amide I and Amide II vibration coupled modes; for the principal Amide I mode and for the Amide II vibration coupled modes, we estimate a wavenumber red-shift of 9 cm^−1^ and 10 cm^−1^, respectively. The estimated EFs range from 3 × 10^4^ to 7 × 10^4^, thereby enabling the detection of spectral variation in the vibrational bands even for a very small number of molecules (3.33 fmol in our experiment). Unlike many traditional methods, also based on radioactive compounds, our technology enables label-free and real-time monitoring, thus providing inherent advantages, including cost-effectiveness. It is also worth remarking that, as demonstrated in previous studies [36], our proposed platform is reusable, since the functionalized MS can be regenerated via a well-established chemical treatment so as to restore the original plasmonic resonance. Finally, we highlight that the red-shifts observed in the Amide I mode and Amide II vibration coupled modes are due to the breaking of hydrogen bonds that are responsible for the secondary and tertiary protein structure, and so they are present in all proteins. Therefore, our proposed device can be exploited to monitor the denaturation process of any protein.

In conclusion, SEIRA spectroscopy can be a powerful technique for investigating changes in the interaction between chemical groups that lead to the functionality of proteins. Moreover, via chemical modifications of the gold surface, it is possible to bind the lipid membranes too, thereby allowing experiments on biological dynamics of more complex processes [65]. This technology holds great promise to outperform modern surface spectroscopic techniques, with ample room for improvement as an analytical tool for biological samples and processes.

## Figures and Tables

**Table 1:** Experimental geometrical dimensions of each NA constituting the different pixels of our proposed MS, and corresponding experimental and numerical resonant wavenumbers.

	Geometrical Parameters			ν_exp_ (cm^−1^)	ν_num_ (cm^−1^)
	*L* (nm)	*W* (nm)	*p* (µm)		
Pixel #1	1117	255	2.0	1500	1457.5
Pixel #2	1247	267	1.5	1600	1601.11
Pixel #3	1027	270	1.5	1737	1834.5
Pixel #4	843	250	1.5	2032	1945

## Data Availability

Not applicable.
